# Erythromycin Shortage in an NTM‐Endemic Country: Implications for Bronchiectasis Care and Antimicrobial Stewardship

**DOI:** 10.1002/resp.70189

**Published:** 2025-12-14

**Authors:** Takanori Asakura, Ho Namkoong

**Affiliations:** ^1^ Division of Pulmonary Medicine, Department of Medicine Keio University School of Medicine Tokyo Japan; ^2^ Department of Infectious Diseases Keio University School of Medicine Tokyo Japan

**Keywords:** chronic airway disease, exacerbations, macrolide therapy, mycobacterium avium complex (MAC) lung disease, nontuberculous mycobacteria (NTM) pulmonary disease

Long‐term macrolide therapy is now an established strategy to prevent exacerbations in patients with non‐cystic fibrosis bronchiectasis (NCFB). Three pivotal randomised controlled trials in the 2010s [1, 2, 3], using azithromycin or erythromycin, consistently demonstrated reductions in exacerbation frequency and improvements in patient‐reported outcomes and pulmonary function tests [4]. These data informed international guidelines, which now recommend long‐term macrolides as a key option for selected bronchiectasis patients [5, 6]. However, how this evidence is implemented is strongly influenced by local epidemiology and drug availability. In the Asia‐Pacific region, Japan is a striking example: it has one of the highest incidences of nontuberculous mycobacterial (NTM) pulmonary disease (NTM‐PD) worldwide and a long tradition of using macrolides for chronic airway diseases. In this context, the recent disruption of erythromycin (Erythrocin) supply has had a disproportionate clinical impact and has exposed a vulnerability in both local practice and the global antibiotic supply chain.

In Japan, the incidence of NTM‐PD, particularly that caused by *Mycobacterium avium* complex (MAC), has now surpassed that of smear‐ or culture‐positive tuberculosis [7]. Epidemiological data further suggest a shift in the bronchiectasis landscape, with post‐tuberculosis disease in men declining and NTM‐associated bronchiectasis in women becoming increasingly prominent [8, 9]. Macrolides, especially clarithromycin and azithromycin, are the backbone of NTM treatment, but only when used as part of appropriate combination regimens. Macrolide monotherapy in patients with unrecognised NTM disease is a well‐known driver of macrolide‐resistant MAC [10], a phenotype that is extremely difficult to treat and associated with poor outcomes [11].

For this reason, international bronchiectasis guidelines emphasise NTM screening before starting long‐term macrolides and warn against macrolide monotherapy in the presence of NTM [5, 6]. Japan also has a uniquely long and influential history with macrolide therapy. The archetypal example is diffuse panbronchiolitis (DPB), once a fatal disease that became a chronic, manageable condition after the introduction of long‐term, low‐dose erythromycin [12]. This therapeutic “macrolide revolution” in DPB is one of the clearest demonstrations that macrolides exert profound immunomodulatory and anti‐inflammatory effects independent of their antimicrobial action. The success in DPB encouraged Japanese clinicians to extend low‐dose macrolide therapy to other chronic airway diseases, including bronchiectasis and chronic rhinosinusitis, well before the large randomised trials were published. Consistent with this history, Japan had the highest baseline rate of long‐term macrolide use in the global phase 3 ASPEN trial [13]. Japanese expert opinion has pushed this risk‐avoidance strategy even further, and long‐term azithromycin or clarithromycin is often withheld in patients with chronic airway disease when NTM cannot be confidently excluded. Instead, erythromycin has been preferred because erythromycin monotherapy, at least in retrospective series of MAC pulmonary disease (MAC‐PD), does not appear to select for clarithromycin‐resistant MAC and is therefore considered less likely to compromise future clarithromycin‐based regimens [14]. Moreover, a propensity score analysis in MAC‐PD suggested that long‐term, low‐dose erythromycin monotherapy may help to suppress disease progression [15]. This is a form of macrolide stewardship shaped by NTM epidemiology. Given that the burden of NTM‐PD is also substantial and rising in many Asian countries beyond Japan [16], similar cautious approaches to long‐term macrolide use are likely to be important across Asia as a whole.

The current erythromycin shortage in Japan therefore creates a unique clinical dilemma. Only clarithromycin is reimbursed for long‐term macrolide prophylaxis in bronchiectasis, and azithromycin remains off‐label in this setting, further constraining treatment choices. If clinicians switch patients to long‐term azithromycin or clarithromycin, they may obtain the anti‐exacerbation benefit shown in randomised trials but at the price of a higher perceived risk of selecting macrolide‐resistant NTM in an NTM‐endemic population. If they forgo macrolide prophylaxis altogether, they risk more frequent exacerbations and potential lung function decline in vulnerable bronchiectasis patients. In practice, many physicians are uncomfortable with both options and instead re‐evaluate the indication on a case‐by‐case basis. Additionally, the risk–benefit balance may differ between settings. In most Western countries, the main concerns related to macrolide therapy in bronchiectasis are gastrointestinal intolerance, QT prolongation, hearing impairment, and the selection of macrolide‐resistant respiratory pathogens, as highlighted in major guidelines and reviews of long‐term macrolide therapy in chronic respiratory disease [5, 6]. In regions with a high burden of NTM‐PD, such as Japan, clinicians and recent reviews place additional emphasis on the risk of inducing macrolide‐resistant NTM in patients with unrecognised or undertreated NTM‐PD who are exposed to long‐term macrolide therapy for chronic airway disease [17].

The immediate cause of the erythromycin shortage in Japan has been attributed, in official notices from the marketing authorisation holder, to manufacturing problems at overseas contract production sites rather than to antimicrobial resistance or newly identified safety concerns. These notices describe shipment suspension and subsequent “limited shipment” of multiple Erythrocin formulations, including tablets, paediatric dry syrups, and intravenous preparations [18]. In response, a joint statement by the Japanese Respiratory Society, Japanese Association for Infectious Diseases, and Japanese Society of Chemotherapy has highlighted the ongoing instability of erythromycin supply and called for prioritisation and careful indication of macrolide use in bronchiectasis [19].

In clinical practice, the erythromycin shortage in Japan has removed what had become a practical option for long‐term macrolide therapy in bronchiectasis and has required clinicians and pharmacists to manage with limited stock, substitute alternative formulations, and repeatedly reassess indications on a case‐by‐case basis. A similar situation was observed during the critical national shortage of cefazolin in Japan, where the loss of a first‐line agent led to increased use of broader‐spectrum parenteral alternatives and raised concerns about adverse effects on antimicrobial stewardship and resistance [20]. A recent systematic review of antibiotic shortages likewise reported that most shortages are driven by economic factors and vulnerabilities in manufacturing capacity rather than by antimicrobial resistance or new safety signals, and that common responses include substitution with less appropriate agents, delays in care, and disruption of stewardship programs [21].

The Japanese experience with erythromycin highlights an often overlooked aspect of antimicrobial stewardship: maintaining reliable access to older, off‐patent antibiotics that continue to have important roles in defined clinical settings. Erythromycin is no longer the preferred agent for many acute infections, yet it remains relevant for modulation of chronic airway disease, as a prokinetic drug, and in certain patient groups. For these indications, a prolonged interruption of supply is effectively similar to losing the drug because of resistance. This situation is difficult to reconcile with the World Health Organization essential medicines framework, which states that essential medicines should be continuously available, affordable, and of assured quality within functioning health systems [22].

For clinicians managing bronchiectasis in an NTM‐endemic country such as Japan, erythromycin has long provided a way to obtain the benefits of long‐term macrolide therapy while limiting the perceived risk of selecting macrolide‐resistant NTM. The ongoing shortage removes this option and forces difficult choices: to accept a higher risk of exacerbations and possible lung function decline, or to use alternative macrolides that may carry a higher risk of promoting resistance in unrecognised NTM disease. This is not a theoretical problem but a recurrent issue in outpatient clinics and hospitals. As strategies for long‐term macrolide use in bronchiectasis are refined, the Japanese case suggests that macrolides are not freely substitutable across all clinical contexts and that maintaining a stable supply of older agents such as erythromycin should be regarded as part of the broader responsibility to preserve access to essential antimicrobial therapies.

## Funding

This work was supported by grants from AMED (JP24ak0101206, JP25wm0225037, JP25fk0108673, JP25tm0424232), JST PRESTO (JPMJPR21R7), JSPS KAKENHI (23H02952, 24KK0158, 25K02695) and Takeda Science Foundation.

## Conflicts of Interest

Takanori Asakura has consulted for Insmed, Boehringer Ingelheim, and CSL Behring. Ho Namkoong has consulted for Insmed.
